# Supplementary material for: To tweet or not to tweet, that is the question: A randomized trial of Twitter effects in medical education
Source: PLoS One. 2019 Oct 16;14(10):e0223992. doi: 10.1371/journal.pone.0223992 (PMC6795488; doi:10.1371/journal.pone.0223992)
Supplement: Attachment — Submitted filename: R2R.docx [file pone.0223992.s001.docx]

| General Comments | Author Response | Location |
| --- | --- | --- |
| Reviewer #1 |  |  |
| Authors state that: “We excluded articles published after 2015 because AM has a policy to tweet all new articles, and the journal did not want to disenfranchise authors of articles that were not tweeted as part of a study”. The same applies to placebo in clinical trials but does not prevent from doing placebo-controlled trials. It would be good if authors comment on that. E.g., three-year old articles might attract less attention independently from Tweets. | In our limitations section, we acknowledge and discuss that the age of the articles used in this study may have negatively affected user engagement, including resulting in less attention. We also note in the limitation, however, that “…the tweets created in this study did not reference the age of the article, and so Twitter users would have no way of knowing these were 2015 articles until they clicked on the article link (unless, of course, they were already familiar with the article title). Nonetheless, more work is needed to investigate the impact of tweeting on contemporary articles.” | Pg. 11 Lines 244-50 |
| Authors based the sample size calculation on a difference of 6 views that corresponds to 40-65% effect size based on 30-day HTML Full-Text Views and >100% effect size based on 30-day PDF Full-Text Views. As such, the study is underpowered and the lack of statistical significance is not surprising, especially for Day 1, Day 7 and PDF Full-Text Views. Authors should consider expanding the sample or, at least, carefully commenting on this in the Discussion (and toning it seriously down). Indeed, the only statistically significant measure is the one with reasonable effect size (40% for 30-day HTML Full-Text Views). | We appreciate this critique regarding the sample size. Since the methodology utilized a case-control study, it is unfeasible to expand the sample size with the study. It is imperative to note that the baseline statistics for Twitter activity of the manuscripts is relatively low. Had the sample size been increased, any additional statistically significant findings would have been due to fractions of a difference in Twitter activity between the case and control samples. We made a conscious decision to balance practically significant findings with statistically significant findings. We have updated the discussion to acknowledge the lower sample size. | Pg. 12 Lines 281-285 |
| It would be good to known the number of citations of included articles in the two groups. | We have added details regarding the average number of citations for articles in both the control and experimental arms of the study. We also added a note to our methods section that this data was collected from Web of Science. | Pg. 6 Lines 144-45  Pg. 7 Lines 163-65. |
| Authors might find interesting commenting on Eysenbach G, J Med Internet Res 2011, and Cardona-Grau D et al, Eur Urol Focus 2016. | Thank you for these suggested articles. We have now integrated Cardona-Grau’s work into the discussion section. | Pg. 10 Lines 221-24 |
| Reviewer #2 |  |  |
| Although the comparison between the two groups show statistically difference the authors should better define the type of article that have been downloaded (i.e. original papers , case reports short communications, review articles) in relation to the different times of observation. | We describe the publication types for all articles included in this study as defined by the journal and report the number of article types per arm. We have added a note to our limitations section acknowledging that we did not analyze the impact of publication type and recommend that this question be addressed in future research. | Pg. 7 Lines 157-61  Pg. 12 Lines 256-59 |
| Moreover, the authors should clarify why they conducted the study in the 2018 but the article tweeted referred to 2015. | In the methods section, we acknowledge and discuss that the age of the articles used in this study, which unfortunately was due to journal policies. We also note in our limitations section that the use of older articles may have negatively affected user engagement and is a limitation of the study. | Pg. 4 Lines 94-8 ;  Pg. 11 Lines 244-50. |
| One limitation of the study which should be discussed in the paper is the selection of the tweet indicating only the title of the article. A more appealing choice probably could increase the attention of followers. | We recognize that tweeting only the title of the article is a limitation. We have added additional language to this acknowledgment. | Pg. 11 Lines 246-51 |
